# Preoperative assessment of tumor eloquence and resectability: an international survey

**DOI:** 10.1007/s11060-025-05067-0

**Published:** 2025-05-21

**Authors:** Emma Rammeloo, Jacob S. Young, Joost W. Schouten, Eelke M. Bos, Shawn L. Hervey-Jumper, Christine Jungk, Sandro M. Krieg, Timothy Smith, Jordina Rincon-Torroella, Chetan Bettegowda, Takashi Maruyama, Arthur Wagner, Philippe Schucht, Marike L. D. Broekman, Steven De Vleeschouwer, Brian V. Nahed, Mitchel S. Berger, Arnaud J. P. E. Vincent, Jasper K. W. Gerritsen

**Affiliations:** 1 Department of Neurosurgery, Erasmus Medical Center, Dr. Molewaterplein 40, 3015 GD Rotterdam, The Netherlands; 2 Department of Neurosurgery, University of California, San Francisco, CA USA; 3 Department of Neurosurgery, University Hospital Heidelberg, Heidelberg, Germany; 4 Department of Neurosurgery, Brigham and Women’s Hospital, Boston, MA USA; 5 Department of Neurosurgery, Johns Hopkins University, Baltimore, MD USA; 6 Department of Neurosurgery, Tokyo University, Tokyo, Japan; 7 Department of Neurosurgery, Technical University Munich, Munich, Germany; 8 Department of Neurosurgery, Inselspital University Hospital Bern, Bern, Switzerland; 9 Department of Neurosurgery, Haaglanden Medical Center, and Leiden University Medical School, The Hague, The Netherlands; 10 Department of Neurosurgery, University Hospitals Leuven, Leuven, Belgium; 11 Department of Neurosurgery, Massachusetts General Hospital, Boston, MA USA

**Keywords:** Glioma, Eloquence, Functional areas, Neuro-oncology, Neurosurgery, Survey, Decision-making

## Abstract

**Background and objectives:**

Tumor location and its proximity to eloquent brain areas are key factors in glioma surgery decision-making. However, the absence of a consensus definition for eloquent brain areas leads to variability in surgical decision-making. This survey aimed to assess this heterogeneity in defining eloquent brain regions.

**Methods:**

A survey was distributed among neurosurgeons in the United States, Europe, Latin America, and Australasia between February and November 2023. Respondents rated the eloquence of various brain structures on a Likert scale and reported their use of preoperative techniques. Twelve glioma and glioblastoma cases were presented to assess opinions on tumor location eloquence and preferred surgical approaches.

**Results:**

157 neurosurgeons from 25 countries responded to the survey. Two-thirds (68%) agreed on the need for a standardized definition of eloquence, while only 23% applied existing eloquence grading scales. Eloquence ratings varied, with the highest variation reported for the corona radiata, uncinate fasciculus and superior longitudinal fasciculus. In patient cases, variability was observed at four levels of decision-making: (1) degree of eloquence; (2) preferred surgical modality; (3) use of intraoperative mapping; (4) the preferred mapping modality (asleep or awake).

**Conclusions:**

This survey highlights the variability in defining eloquence and its impact on glioma surgery decision-making. This lack of consensus limits the reliability of eloquence as a descriptor of tumor location, affecting patient care and comparability across studies. Future research should focus on the development of an easy-to-use, objective method (based on intraoperative data) for identifying eloquent brain regions preoperatively.

**Supplementary Information:**

The online version contains supplementary material available at 10.1007/s11060-025-05067-0.

## Introduction

Currently, the standard-of-care for high-grade glioma consists of surgery followed by adjuvant chemotherapy and radiotherapy [1–3]. The neurosurgical approach to these tumors—including preoperative imaging techniques, extent of resection, and the application of intraoperative mapping—is influenced by the surgeon’s experience, patient-related factors (age, performance, comorbidities), and tumor-related factors (grade, size, location, relationship to functional parenchyma) [4–7]. A lack of clinical guidelines based on objective data has resulted in large variability in neurosurgical decision-making between surgeons and centers [8–10]. One important factor in the decision-making for glioma treatment that is prone to variability and subjectivity is eloquence of the tumor location [11]. Eloquence in this context refers to brain regions “that speak to readily identifiable neurological function and, if injured, result in a disabling neurological deficit” [12]. A central challenge in glioma and glioblastoma surgery is achieving an optimal on co-functional balance—maximizing resection while preserving neurological function. Intraoperative mapping is the gold standard for maintaining this balance, with cortical and subcortical eloquence serving as key determinants for its use [1, 13]. The current literature on gliomas describes the primary motor and sensory cortices, cortical language areas [dominant frontal operculum (Broca’s area) and dominant superior temporal gyrus (Wernicke’s area)], visual cortex, internal capsule (including corticospinal tracts and sensory white matter pathways), basal ganglia, and the thalamus as eloquent regions [14]. However, for many other structures the “degree” of eloquence is more ambiguous, such as the supplementary motor cortex (SMA), insula, corpus callosum, hippocampus, and subcortical white matter tracts [14]. Notably, a recent study on the microsurgical management of cerebral cavernomas identified functional eloquence in structures traditionally classified as non-eloquent [15]. Moreover, various imaging methods and classification systems exist for preoperative localization of eloquence [16], yet none are entirely satisfactory for objective, preoperative decision-making in glioma treatment [14]. This variability in preoperative localization and definition renders eloquence unsuitable as an objective and reliable preoperative factor. Consequently, it may result in suboptimal treatment for certain patient subgroups [1] and hinder reliable comparisons between glioma patients and cohorts, thereby impeding large multicenter studies crucial for enhancing glioma care.

A systematic review [14] recently conducted by our team provided a qualitative benchmark assessment of the current definition of eloquence in neurosurgery. However, synonyms, incomplete definitions, and variations in extensiveness of definitions hindered an objective evaluation of this definition. Therefore, this survey aims to objectively assess global heterogeneity in defining eloquence and to analyze the results based on geographical location, experience, and resources. The results will provide information on the perceived degree of eloquence of different structures, the use of preoperative techniques, and surgeons’ approaches to various patient cases. By identifying factors potentially underlying the variability in preoperative decision-making, the findings will ultimately aid in the development of a new, objective preoperative grading system for eloquence. This could aid neurosurgeons in their preoperative decision-making to improve surgical outcomes on an individual patient level and enhance the comparability of patients and cohorts.

## Methods

### Survey design

The primary aim of the survey was to assess the degree of eloquence that individual neurosurgeons assign to different anatomical structures and how this influences their preferred surgical approaches in patient cases. The anatomical structures were selected based on definitions of eloquence in existing literature, as identified in our systematic review [14]. Patient cases were selected based on tumor involvement of these anatomical structures. Respondents were asked to classify tumor locations as eloquent, near eloquent, or not eloquent, and to select a preferred surgical approach. Options included no surgery, biopsy, decompression/partial resection, maximal safe resection with asleep mapping or monitoring, maximal safe resection with awake mapping or monitoring, and maximal safe resection without mapping or monitoring. For analysis, surgical approaches were grouped into aggressive (maximal safe resection, with or without mapping/monitoring) or less invasive (no surgery, biopsy, decompression/partial resection). Additionally, the response options ‘near eloquent’ and ‘eloquent’ were combined and compared to ‘not eloquent.’ If more than 75% of respondents selected either of these options, the response was considered consensual. The secondary aim was to evaluate the use of preoperative techniques for localizing eloquence, including grading scales and classification systems (the Spetzler–Martin grading system [12], UCSF LGG-scale [17], Sawaya eloquence grading system [18], Shinoda topographical tumor grading system [19], and the Friedlein grading system [20]). The survey targeted neurosurgeons with various levels of professional experience: neurosurgical consultants with over ten years of experience since completing their fellowship, neurosurgical consultants with five to ten years of experience since completing their fellowship, neurosurgical consultants with less than five years of experience since completing their fellowship, neurosurgical fellows, and neurosurgical residents. Additional baseline characteristics were gender, age, country and WHO region, affiliation, the number of glioma resections performed, and the number of awake craniotomies performed. Affiliated institutes were categorized as academic or university hospitals, non-academic or community hospitals, private institutions, and others.

### Statistical analyses

On 11 November 2023, LimeSurvey data were exported into an Excel file for further analysis using *R* version 4.3.2 (the *R* foundation, Vienna, Austria). Data were grouped according to the baseline characteristics region (US or Europe), affiliation, number of glioma resections performed, and number of awake craniotomies performed. To analyze overall response differences the *χ*^2^ test for proportions with the Marascuillo procedure and Bonferroni correction for multiple testing were used. These same statistical tests were applied to analyze differences in survey responses based on the surgeon’s experience (expressed as number of glioma resections and awake craniotomies performed). Further subgroup analysis of categorical survey responses was performed with multivariate (logit) regression with type of institution and region (United States/Europe) as independent variables. For variables with > 2 response options, dummy coding was used for processing responses into dichotomous variables. Multinominal linear regression was used to analyze continuous survey outcomes. Statistical significance was set at 5%. The data was analyzed using available-case analysis.

### Survey dispersal

Using Mailchimp (Atlanta, GA, USA), a link to the online LimeSurvey questionnaire platform (LimeSurvey GmbH, Hamburg, Germany) was distributed to electronic mailing lists of the Congress of Neurological Surgeons (CNS) and the Dutch Neurosurgical Association (NVvN). Additionally, the survey was distributed by mail among members of the British Society of Neurological Surgeon (SBNS) and included in the monthly newsletter of the European Association of Neurological Societies (EANS), the American Association of Neurological Surgeons (AANS), the Latin American Federation of Neurosurgical Societies (FLANC), the Neurosurgical Society of Australasia (NSA), the Asia Australasian Society of Neurological Surgeons (AASNS), and the World Federation of Neurosurgical Societies (WFNS). Participation in the survey was voluntary and anonymous, and participants were not offered any compensation. The survey was open for entries between February and November 2023 and was carried out by the PIONEER Consortium [21].

## Results

A summary of the baseline characteristics of respondents is displayed in Table 1. A total of 157 responses (response rate 2.15%) was received from 25 different countries (absolute number of respondents in brackets): Armenia (1), Australia (2), Belgium (2), Brazil (3), Canada (6), Chile (2), Costa Rica (1), Croatia (2), Denmark (1), Dominican Republic (1), Egypt (1), France (1), Germany (5), India (6), Iraq (1), Israel (1), Italy (6), Moldova (1), Philippines (1), Saudi Arabia (1), The Netherlands (6), Turkey (2), United Arab Emirates (2), United Kingdom (25), United States of America (75). The majority of respondents were male (90%, n = 141), originated from the United States/Canada (52%, n = 81) or Europe (34%, n = 53), were employed in academic practices/university hospitals (67%, n = 105), and were consultant neurosurgeons with more than ten years of experience since completing their fellowship (64%, n = 100). Experience with glioblastoma resections varied: 26% had performed less than 100 resections (n = 41), 37% had performed 100–500 resections (n = 58), and another 37% had performed more than 500 glioblastoma resections in their career (n = 58). Most respondents had performed less than 100 awake craniotomies during their career as a neurosurgeon (80%, n = 125).

**Table 1:** Baseline characteristics of respondents

Characteristic	Number of responses (%) (*n* = 157)
Gender	
Male	141 (89.8)
Female	16 (10.2)
Region (World Health Organization)	
American Region—United States/Canada	81 (51.6)
American Region—Latin America	7 (4.5)
European Region	53 (33.8)
Eastern Mediterranean Region	5 (3.2)
South-East Asia Region	1 (0.6)
Western Pacific Region	4 (2.5)
African Region	0 (0)
Institute	
Academic practice/University hospital	105 (66.9)
Non-academic practice/Community hospital	29 (18.5)
Private practice	17 (10.8)
Other	6 (3.8)
Training level	
Consultant neurosurgeon < 5 years of experience	24 (15.3)
Consultant neurosurgeon, 5–10 years of experience	17 (10.8)
Consultant neurosurgeon, > 10 years of experience	100 (63.7)
Neurosurgical fellow	5 (3.2)
Neurosurgical resident	11 (7)
Total number of GBM resections performed	
< 100	41 (26.1)
100–500	58 (36.9)
> 500	58 (36.9)
Total number of awake craniotomies performed	
< 50	81 (51.6)
50–100	44 (28)
100–500	24 (15.3)
> 500	8 (5.1)

### General questions on eloquence

**Fig. 1:**
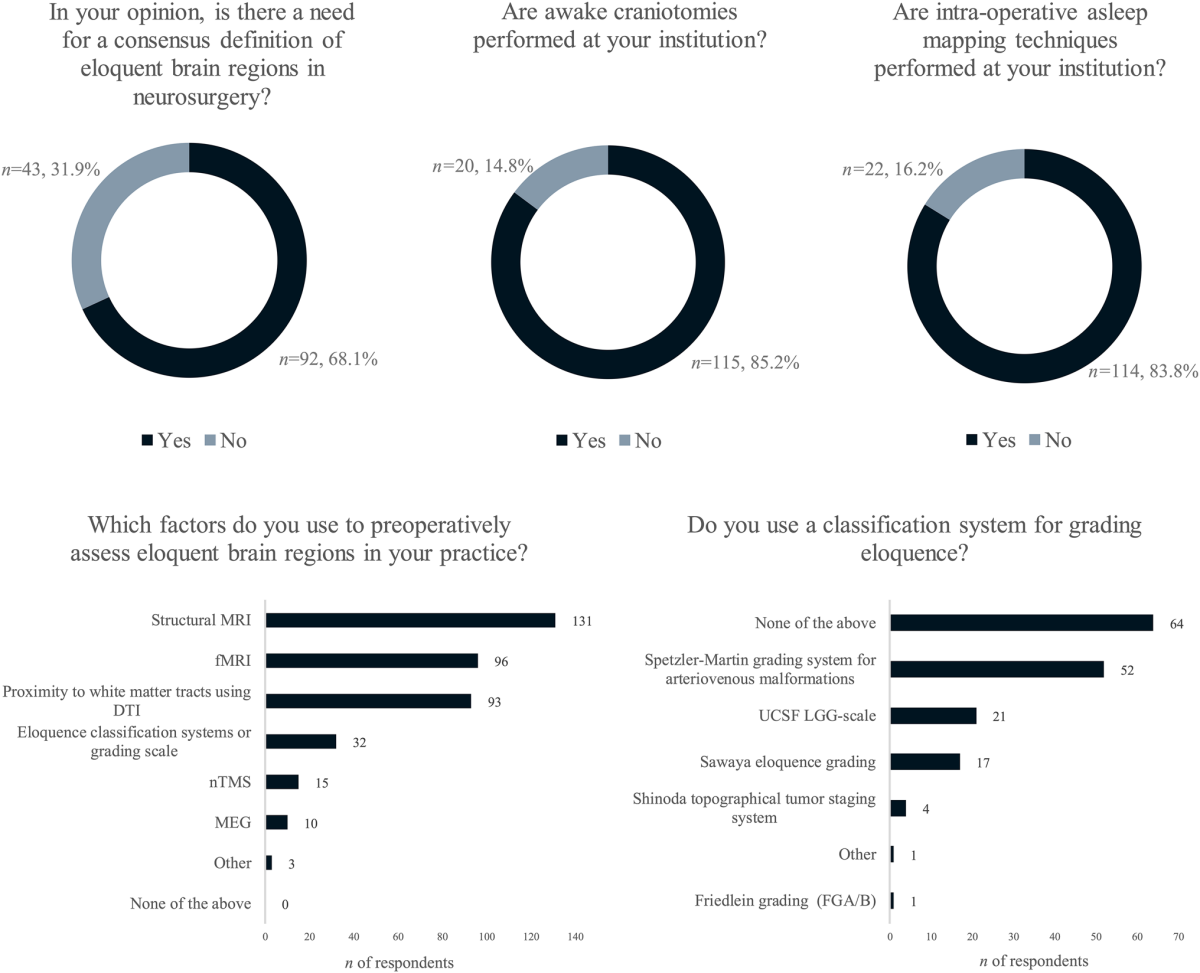
General questions on eloquence

The second part of the survey assessed respondents’ experience with intraoperative mapping (both awake and asleep) and methods to localize eloquence preoperatively. These results are visualized in Fig. 1 and displayed in Table 2. Two-thirds of the respondents stated that a consensus definition of eloquent brain regions in neurosurgery is required (n = 92, 68%). Most respondents (78%, n = 105) reported the use of both awake and asleep intraoperative mapping at their institutions. Nearly all respondents indicated that structural MRI is employed as a pre-operative mapping technique to localize eloquent brain regions. However, only 23% (n = 32) reported utilizing eloquence classification systems or grading scales, with the Spetzler–Martin grading system for arteriovenous malformations [12] being the most commonly selected.

**Table 2:** General questions on eloquence

Question	Response options	Overall response (%)
In your opinion, is there a need for a consensus definition of eloquent brain regions in neurosurgery? *n* = 135	• Yes	92 (68.1)
	• No	43 (31.9)
Are awake craniotomies performed at your institution? *n* = 135	• Yes	115 (85.2)
	• No	20 (14.8)
Are intra-operative asleep mapping techniques performed at your institution? (Evoked potentials (MEP/SSEP) with subdural grid/strip electrodes or continuous dynamic mapping (CDM) on suction tube or CUSA) *n* = 135	• Yes	113 (83.7)
	• No	22 (16.2)
Which factors do you use to preoperatively assess eloquent brain regions in your practice? (please select all that apply) *n* = 137	• Anatomical location based on structural MRI	131 (95.6)
	• Proximity to white matter tracts using DTI	93 (67.9)
	• Navigated Transcranial Magnetic Stimulation (nTMS)	15 (10.9)
	• Functional MRI (fMRI)	96 (70.1)
	• Magnetoencephalography (MEG)	10 (7.3)
	• Eloquence classification systems or grading scales	32 (23.4)
	• None of the above	0 (0)
	• Other – Neuropsychiatric testing – Physical exam as altered by tumor	2 (1.5) 1 (0.7)
Do you use a classification system for grading eloquence? (please select all that apply) *n* = 137	• Sawaya eloquence grading	17 (12.4)
	• Spetzler–Martin grading system for arteriovenous malformations	52 (38.0)
	• UCSF LGG-scale	21 (15.3)
	• Friedlein grading (FGA/B)	1 (0.7)
	• Shinoda topographical tumor staging system	4 (2.9)
	• None of the above	64 (46.7)
	• Other	
	– NTMS risk stratification	1 (0.7)
	– “Our own one”	1 (0.7)

### Eloquence of various brain structures

**Fig. 2:**
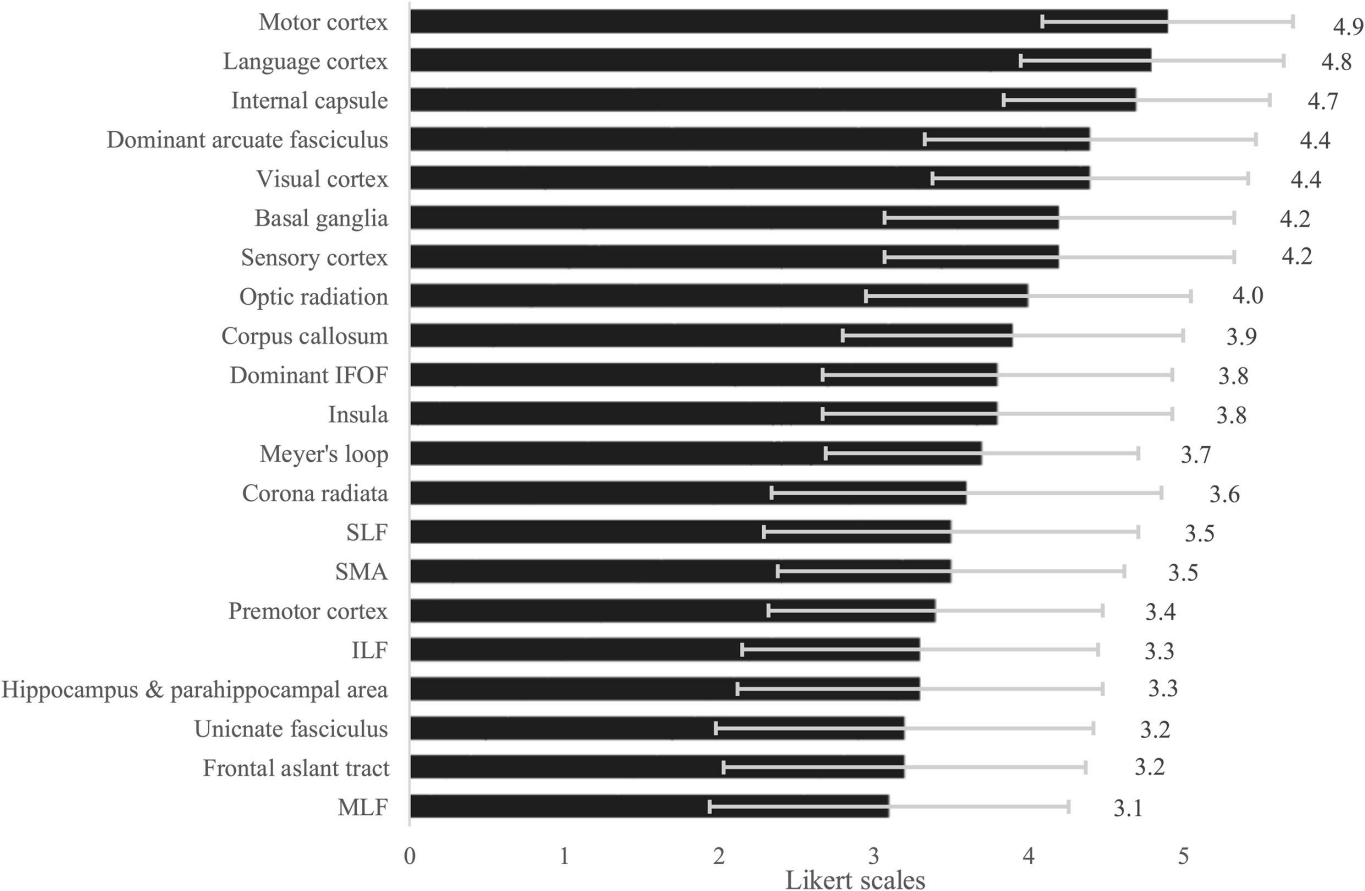
Mean Likert scale ratings of eloquence per anatomical structure

Respondents rated the eloquence of various anatomical structures on a Likert scale, where 1 indicated ‘not eloquent’ and 5 indicated ‘very eloquent’. The results are presented in Fig. 2. The five highest-rated structures were the primary motor cortex (mean 4.8), cortical language areas and internal capsule (mean 4.7), visual cortex, and the dominant arcuate fasciculus (mean 4.4). Conversely, the five lowest-rated structures were the inferior longitudinal fasciculus (ILF) (mean 3.3), hippocampus and parahippocampal area (mean 3.3), uncinate fasciculus and frontal aslant tract (mean 3.2), and the middle longitudinal fasciculus (mean 3.1). The structures with the highest variability in ratings—indicating the least consensus on their degree of eloquence—were the corona radiata (mean 3.6, SD 1.26), uncinate fasciculus (mean 3.2, SD 1.22), superior longitudinal fasciculus (mean 3.5, SD 1.21), hippocampus and parahippocampal area (mean 3.3, SD 1.18), and the frontal aslant tract (mean 3.2, SD 1.17).

### Practical cases

**Fig. 3:**
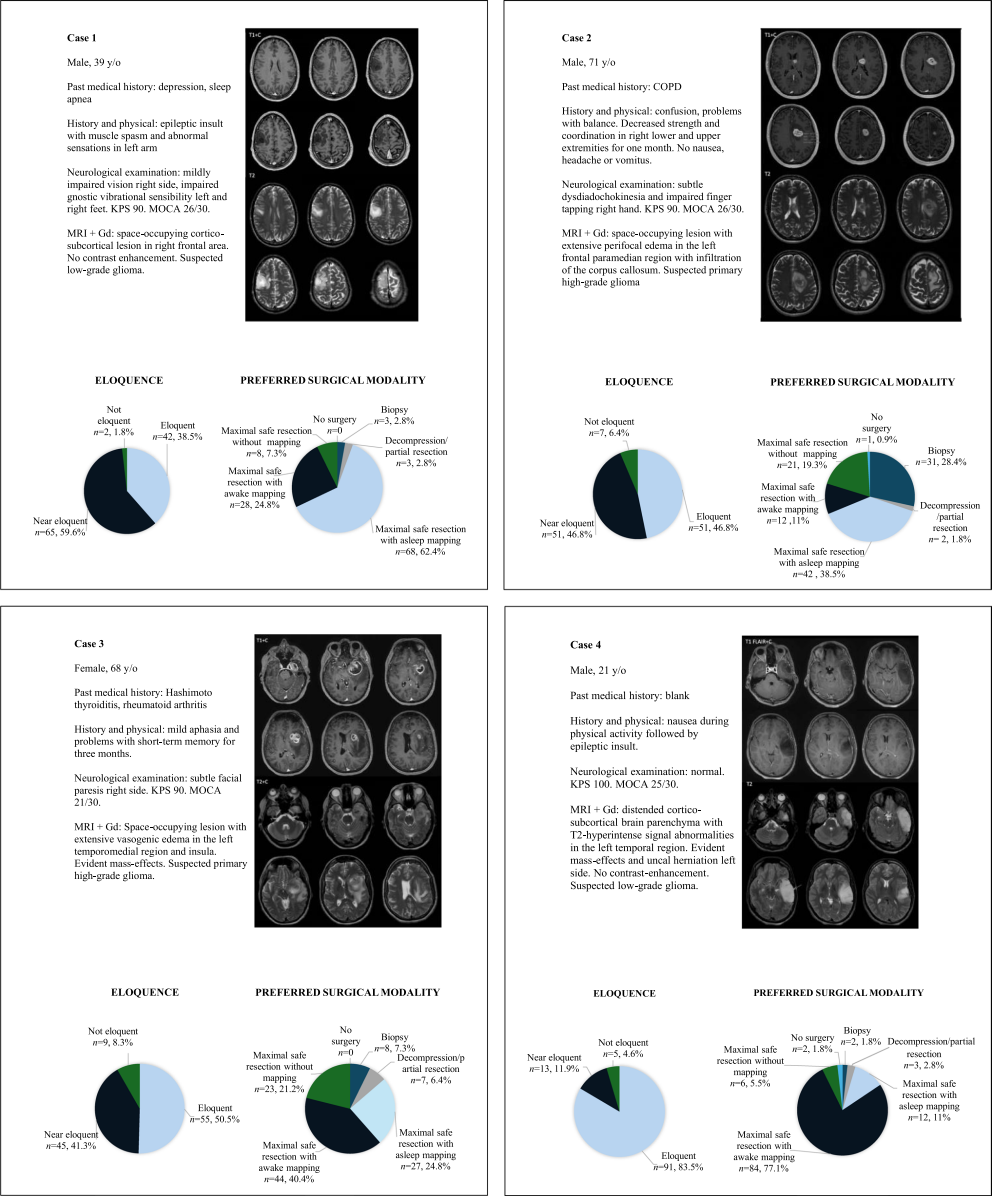

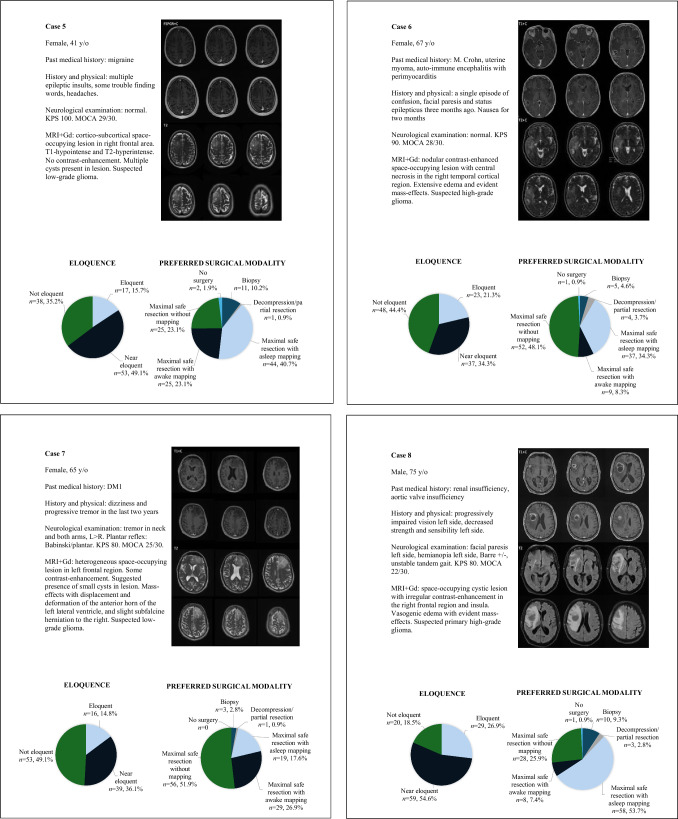

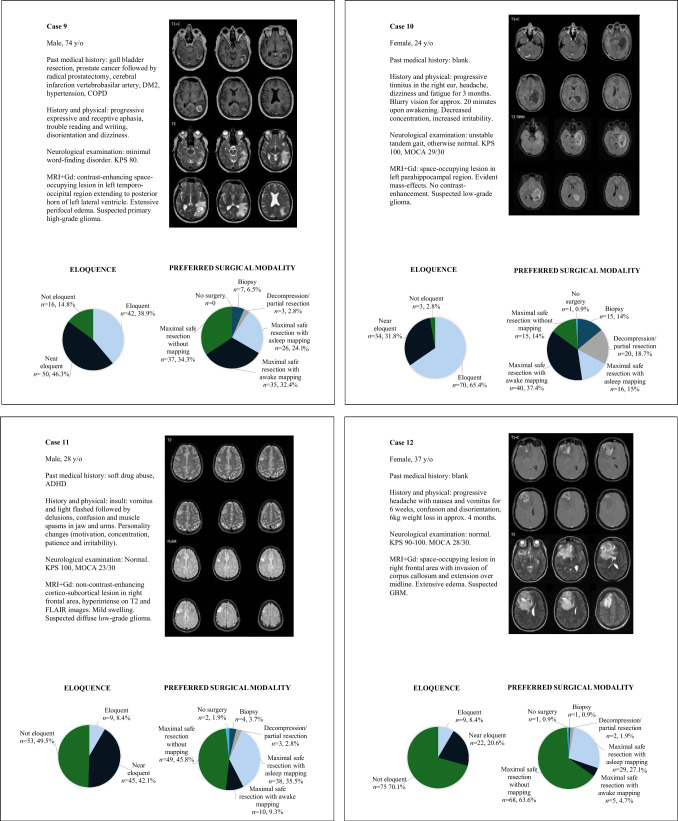
Clinical details of patient cases

**Fig. 4:**
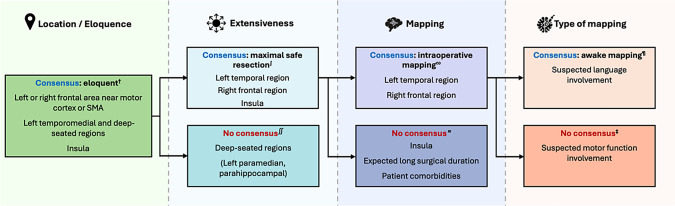
Decision-making consensus in patient cases.This figure visualizes the consensus on four levels of decision-making in the patient cases (from left to right): (1) the degree of eloquence of the tumor location; (2) aggressive versus less invasive approaches; (3) whether to use intraoperative mapping; and 4) the preferred mapping modality (asleep or awake). ^†^Cases 1, 2, 3, 4, 8, 9, 10; ^∫^Cases 1, 3, 4, 8 and 9; ^∬^Cases 2, 10; ^∾^Cases 1, 3, 4; ^≈^Cases 8, 9; Case 4; ^‡^Cases 1 and 3

The results of the questions on the twelve glioma and glioblastoma patient cases are summarized in Supplementary Table 3. The clinical details of each case are described in Fig. 3. Variability in responses was observed on four levels of decision-making: (1) the degree of eloquence; (2) aggressive versus less invasive approaches; (3) whether to use intraoperative mapping; and (4) the preferred mapping modality (asleep or awake). These levels of decision-making are visualized in Fig. 4.

Respondents generally agreed that the tumor locations in Cases 1 (98%), 2 (94%), 3 (92%), 4 (95%), 8 (82%), 9 (85%), and 10 (97%) were eloquent (Fig. 3). Subsequently, the responses in Cases 2 and 10 did not show consensus on an aggressive versus less invasive approach (69% vs. 31% in Case 2, 66% vs. 34% in Case 10). In Cases 1 (95%), 3 (86%), 4 (94%), 8 (87%) and 9 (91%), the majority of respondents preferred to pursue maximal safe resection, with a subsequent preference for intraoperative mapping in Cases 1 (92%), 3 (76%) and 4 (94%). Among respondents who favored an aggressive surgical approach, the use of intraoperative mapping varied more in Case 8 (70%) and Case 9 (62%). Thus, consensus on the first three levels of decision-making was observed in three cases (Cases 1, 3 and 4). Consensus on the fourth level was only observed in Case 4: among respondents who favored an aggressive surgical approach with intraoperative mapping, most preferred maximal safe resection with awake mapping or monitoring (88%), and only a small proportion preferred asleep mapping or monitoring (12%). In Cases 1 and 3 this consensus was not as clear. Within the group favoring an aggressive surgical approach with intraoperative mapping, 71% of respondents favored asleep mapping in Case 1 and 29% of respondents favored awake mapping. In Case 3, 38% of respondents favored asleep mapping and 62% of respondents opted for awake mapping.

No consensus on the degree of eloquence was observed in cases 5, 6, 7, 11 and 12 (Figs. 3, 4). Nevertheless, an aggressive surgical approach was favored in all these cases—91% in Case 6, 96% in Case 7, 91% in Case 11, and 95% in Case 12. Subsequently, within the group favoring an aggressive surgical strategy no consensus was reached on the preference for intraoperative mapping in these cases, with reported use at 73%, 47%, 46%, 50% and 33%, respectively.

### Multivariate subgroup analyses

Subgroup analyses of survey responses were carried out for the independent variables type of institution (academic vs. non-academic) and region (US vs. Europe). Multivariate subgroup analyses revealed few significant differences. The results are displayed in Supplementary Tables 2–5.

## Discussion

### Key results

This survey aimed to objectively assess global heterogeneity in defining eloquence and consequent surgical modalities and to analyze results by geography, experience, and resources. While the results showed a general consensus on which structures are considered eloquent, substantial variability was observed in preoperative methods employed to localize eloquence and in respondents’ views on tumor location and surgical strategies across most patient cases. Uniformity in decision-making in a practical setting was observed in only one patient case.

### Interpretation of results

Approximately half of the respondents reported not to use an existing system to grade eloquence, despite two-thirds agreeing on the need for a consensus definition of eloquent brain. Of the existing classification systems, the Spetzler–Martin grading system was selected by most respondents, despite being developed for arteriovenous malformations and not for gliomas [12]. This underlines the clinical need for the development of an objective grading scale for eloquence in glioma surgery. Moreover, significant heterogeneity was observed in preoperative imaging methods (e.g., structural MRI, DTI, fMRI). However, there was reasonable agreement on which anatomical structures are eloquent. The highest-rated structures—primary motor cortex, cortical language areas, internal capsule (including corticospinal tracts and sensory white matter pathways), visual cortex, and dominant arcuate fasciculus—align with the literature. Exceptions to this are the internal capsule and dominant arcuate fasciculus, which are rarely included in clinical study criteria but were highly rated in this survey.

In the practical part of the survey, preoperative decision-making in 12 glioma and glioblastoma cases was assessed on four levels: (1) tumor location eloquence, (2) aggressive versus less invasive approach, (3) use of intraoperative mapping, and (4) preferred mapping modality (awake or asleep). The results revealed variability in consensus across all of these levels. These levels of decision-making are visualized in Fig. 4.

At the first level, consensus on tumor location eloquence was observed for tumors in the left or right frontal area near the motor cortex or SMA, left temporomedial and deep-seated regions, and insula (Cases 1, 2, 3, 4, 8, 9, and 10)—aligning with the highest-rated eloquent structures in the theoretical survey. In contrast, for tumors in right frontal or temporal regions or left frontal regions further from the ‘classic’ eloquent areas, the results showed little agreement on eloquence (Cases 5, 6, 7, 11, and 12).

At the second level, the preferred surgical modalities were divided into two categories: less invasive strategies (biopsy or partial resection) and aggressive strategies (maximal resection with or without intraoperative mapping). In most cases in which the tumor location was deemed eloquent, aggressive resection was the preferred strategy (Cases 1, 3, 4, 8 and 9). However, for deep-seated tumors, such as in Cases 2 and 10, opinions were divided between less invasive and aggressive strategies, suggesting that eloquent locations may be categorized into subgroups requiring distinct strategies.

In cases where the tumor location was deemed eloquent and aggressive surgery was preferred, consensus on the third level was observed only in Cases 1, 3 and 4, with most respondents opting for the use of intraoperative mapping. In cases 8 and 9, a substantial proportion of respondents selected maximal safe resection without the use of intraoperative mapping, even though they aim to aggressively operate on an eloquent tumor. This could be due to tumor location or expected surgical duration (Case 8, insula), or patient comorbidities (Case 8 and 9). For eloquent tumors with a preference for aggressive surgery with intraoperative mapping, asleep mapping is often preferred for motor-eloquent tumors (Case 1, right frontal cortico-subcortical lesion). When language eloquence is suspected (Case 4, left temporal cortico-subcortical lesion; Case 3, left temporomedial lesion), awake mapping is more frequently chosen. In cases lacking consensus on eloquence (Cases 5, 6, 7, 11, and 12), aggressive strategies were generally favored. No clear preference for intraoperative mapping emerged in these cases, though the proportion of respondents favoring its use aligned with those who considered the tumor location eloquent or near eloquent.

### Strengths and limitations

An inherent limitation of survey studies is sampling bias. Despite global distribution, most respondents were from high-income regions (North America and Western Europe). This may affect their available methods and materials, influencing preferences and limiting the generalizability to middle- and low-income countries. Additionally, many questions toward the end of the survey were left unanswered, only 106 respondents completed the survey, and the overall low response rate limited reliable subgroup analyses. These limitations may be a result of the length of the survey and surgeons’ inability to allocate sufficient time to complete it. Moreover, we were unable to correlate the observed response heterogeneity with surgical outcomes. Additionally, we could not explore further how decision-making and the role of eloquence differed between low-grade gliomas and glioblastomas, despite potential differences in onco-functional priorities—e.g. favoring functional preservation in low-grade gliomas and oncological control in glioblastomas. This presents an interesting opportunity for additional (retrospective) studies. Strengths of the study are the global distribution, the combination of theoretical and practical questions, the variability of patient cases, and subgroup analyses comparing academic and non-academic centers as well as US and EU surgeons.

### Comparison with literature

Several studies have investigated surgical decision-making regarding tumor eloquence. Whereas the presented study and a crowdsourcing study [22] highlight variability in choosing aggressive versus less invasive approaches Muller et al. [23] reported general agreement among twelve neurosurgical teams on biopsy and resection decisions, except for tumors in the right superior frontal gyrus and left superior parietal lobe. Similarly, Sonabend et al. [24] found variability in decisions for biopsy, subtotal resection, or gross-total resection for tumors in the left temporal, left insular, and right frontal lobes. These findings align with our results, showing less consensus for tumors in both classic eloquent and ambiguously eloquent locations.

This variability may, in part, reflect limitations in existing tools used to assess resectability. Neurosurgical approaches to gliomas and glioblastomas have been studied in relation to anatomical location, leading to the development of resectability atlases for gliomas [25–27]. In these atlases, residual tumor may be associated with functionally eloquent regions. However, the atlases provide limited information on the intraoperative probability of encountering functional tissue or predicting postoperative deficits. As such, they fall short in guiding decisions between aggressive approaches (e.g., maximal safe resection with or without mapping) and less invasive strategies (e.g., biopsy, decompression).

An additional factor complicating the assessment of eloquence and glioma resectability is the conceptual shift from a localizationist perspective to one centered on network connectivity. We propose that the anatomical structures included in this survey are not intrinsically eloquent, but derive their functional importance from their integration within larger neural networks—the connectome. This broader view of eloquence also encompasses higher-order cognitive functions [15, 28]. While these functions are still underrepresented in current surgical frameworks, we included several cortical regions and subcortical white matter tracts associated with them in the survey. The exploration and intraoperative mapping of higher cognitive functions remain limited, but represent an important direction for future research.

### Conclusion and future directions

This survey highlights the variability in defining eloquence and its impact on preoperative decisions in glioma surgery. It reveals a lack of standardized methods and objective grading scales for preoperative eloquence localization, despite the recognized need for a consensus definition of eloquence. As a result, uncertainty about tumor eloquence and optimal surgical strategies was evident in clinical cases. This lack of consensus applies to all subgroups, as no notable differences emerged from the subgroup analyses based on continent, experience, or institution. Without consensus on eloquence or surgical strategies, eloquence cannot reliably serve as an objective preoperative description of tumor location. Ultimately, no imaging modality replicates real-time mapping information, and tissue functionality can only be confirmed intraoperatively. Therefore, “eloquence” in the preoperative context should refer to the probability of finding a region of interest during the operation rather than describing a location. Using eloquence as a preoperative factor in its current form—prone to variability and subjectivity—may lead to suboptimal care for subgroups of glioma patients and hinder multicenter studies by challenging the comparison of patients and cohorts reliably. Future research should aim to develop an objective and standardized definition and assessment method for eloquence to improve preoperative decision-making in glioma and glioblastoma surgery.

## Supplementary Information

Below is the link to the electronic supplementary material.

Supplementary file1 (PDF 465 kb)

## Data Availability

No datasets were generated or analysed during the current study.
