# Correction to: Preoperative assessment of tumor eloquence and resectability: an international survey

**DOI:** 10.1007/s11060-025-05115-9

**Published:** 2025-07-25

**Authors:** Emma Rammeloo, Jacob S. Young, Joost W. Schouten, Eelke M. Bos, Shawn L. Hervey-Jumper, Christine Jungk, Sandro M. Krieg, Timothy Smith, Jordina Rincon-Torroella, Chetan Bettegowda, Takashi Maruyama, Arthur Wagner, Philippe Schucht, Marike L. D. Broekman, Steven De Vleeschouwer, Brian V. Nahed, Mitchel S. Berger, Arnaud J. P. E. Vincent, Jasper K. W. Gerritsen

**Affiliations:** 1 Department of Neurosurgery, Erasmus Medical Center, Dr. Molewaterplein 40, 3015 GD Rotterdam, The Netherlands; 2 Department of Neurosurgery, University of California, San Francisco, CA USA; 3 Department of Neurosurgery, University Hospital Heidelberg, Heidelberg, Germany; 4 Department of Neurosurgery, Brigham and Women’s Hospital, Boston, MA USA; 5 Department of Neurosurgery, Johns Hopkins University, Baltimore, MD USA; 6 Department of Neurosurgery, Tokyo University, Tokyo, Japan; 7 Department of Neurosurgery, Technical University Munich, Munich, Germany; 8 Department of Neurosurgery, Inselspital University Hospital Bern, Bern, Switzerland; 9 Department of Neurosurgery, Haaglanden Medical Center, and Leiden University Medical School, The Hague, The Netherlands; 10 Department of Neurosurgery, University Hospitals Leuven, Leuven, Belgium; 11 Department of Neurosurgery, Massachusetts General Hospital, Boston, MA USA

**Correction to: Journal of Neuro-Oncology**

10.1007/s11060-025-05067-0

Due to mistakes during typesetting and proof correction, Fig. 3 in this article was partly published in low resolution, and was incomplete, as cases 9–12 were not included.

The original article has been corrected.

