# Supplementary material for: Preoperative assessment of tumor eloquence and resectability: an international survey
Source: J Neurooncol. 2025 May 21;175(1):47–59. doi: 10.1007/s11060-025-05067-0 (PMC12367934; doi:10.1007/s11060-025-05067-0)
Supplement: Supplementary file 1 — Supplementary file1 (PDF 465 kb) [file 11060_2025_5067_MOESM1_ESM.pdf]

Supplementary Table 1: General questions on eloquence

Question	Response options	Overall response (%)	P-value	Adjusted OR for academic vs. non-academic and private practice (95% CI)	P-value	Adjusted OR for European vs. US neurosurgeons (95% CI)	P-value
In your opinion, is there a need for a consensus definition of eloquent brain regions in neurosurgery? *n*=135	• Yes<br>• No	92 (68.1)<br>43 (31.9)	<0.0001*	0.90 (0.41 – 1.96)	0.7825	0.92 (0.42 – 1.99)	0.8313
Are awake craniotomies performed at your institution? *n*=135	• Yes<br>• No	115 (85.2)<br>20 (14.8)	<0.0001*	5.26 (1.92 – 14.43)	0.0013*	0.59 (0.19 – 1.86)	0.3683
Are intra-operative asleep mapping techniques performed at your institution?<br><br>(Evoked potentials (MEP/SSEP) with subdural grid/strip electrodes or continuous dynamic mapping (CDM) on suction tube or CUSA)<br>*n*=135	• Yes<br>• No	113 (83.7)<br>22 (16.2)	<0.0001*	3.21 (1.26 – 8.19)	0.0144*	1.08 (0.31 – 3.77)	0.9045
Please indicate on a Likert scale of 1-5 for each given brain structure whether or not you would classify it as eloquent.	• Motor cortex *(n=135)*	Mean 4.8 (SD=0.81)	NA	Est. 0.26 (SE=0.81)	0.0801	Est. 0.05 (SE=0.88)	0.7612
	• Language cortex *(n=135)*	Mean 4.7 (SD=0.85)		Est. 0.24 (SE=0.85)	0.1207	Est. 0.17 (SE=0.91)	0.3221
	• Internal capsule *(n=134)*	Mean 4.7 (SD=0.86)		Est. 0.29 (SE=0.85)	0.0671	Est. -0.10 (SE=0.93)	0.0514
	• Visual cortex *(n=135)*	Mean 4.4 (SD=1.02)		Est. 0.51 (SE=1.00)	0.0070*	Est. -0.02 (SE=1.09)	0.9076
	• Dominant arcuate fasciculus *(n=134)*	Mean 4.4 (SD=1.07)		Est. 0.57 (SE=1.04)	0.0033*	Est. -0.42 (SE=1.12)	0.0514
	• Sensory cortex *(n=135)*	Mean 4.2 (SD=1.13)		Est. 0.40 (SE=1.12)	0.0562	Est. 0.02 (SE=1.18)	0.9351
	• Basal ganglia *(n=135)*	Mean 4.2 (SD=1.13)		Est. 0.34 (SE=1.13)	0.1034	Est. -0.02 (SE=1.16)	0.9195
	• Optic radiation *(n=134)*	Mean 4.0 (SD=1.05)		Est. 0.45 (SE=1.04)	0.0220*	Est. -0.25 (SE=1.10)	0.2401
	• Insula *(n=135)*	Mean 3.8 (SD=1.13)		Est. 0.11 (SE=1.14)	0.5859	Est. -0.12 (SE=1.16)	0.5787
	• Dominant inferior fronto-occipital fasciculus *(n=134)*	Mean 3.8 (SD=1.13)		Est. 0.39 (SE=1.12)	0.0650	Est. -0.53 (SE=1.16)	0.0188*
	• Meyer's loop *(n=134)*	Mean 3.7 (SD=1.01)					
	• Corona radiata *(n=134)*	Mean 3.6 (SD=1.26)		Est. 0.55 (SE=0.99)	0.0031*	Est. -0.21 (SE=1.05)	0.2981
	• Supplementary motor cortex *(n=135)*	Mean 3.5 (SD=1.12)		Est. 0.49 (SE=1.24)	0.0344*	Est. -0.54 (SE=1.27)	0.0290*
	• Superior longitudinal fasciculus *(n=134)*	Mean 3.5 (SD=1.21)		Est. 0.26 (SE=1.12)	0.2149	Est. -0.56 (SE=1.14)	0.0120*
	• Premotor cortex *(n=135)*	Mean 3.4 (SD=1.08)					
	• Inferior longitudinal fasciculus *(n=134)*	Mean 3.3 (SD=1.15)		Est. 0.44 (SE=1.19)	0.0474*	Est. -0.33 (SE=1.25)	0.1739

	• Hippocampus & parahippocampal area *(n=134)*<br>• Frontal aslant tract *(n=134)*<br>• Uncinate fasciculus *(n=134)*<br>• Middle longitudinal fasciculus *(n=134)*<br>• Corpus callosum *(n=134)*	Mean 3.3 (SD=1.18)<br>Mean 3.2 (SD=1.17)<br>Mean 3.2 (SD=1.22)<br>Mean 3.1 (SD=1.16)<br>Mean 3.0 (SD=1.10)		Est. 0.26 (SE=1.07)<br>Est. 0.47 (SE=1.13)<br>Est. 0.23 (SE=1.18)<br>Est. 0.39 (SE=1.16)<br>Est. 0.37 (SE=1.21)<br>Est. 0.35 (SE=1.15)<br>Est. 0.13 (SE=1.11)	0.2149<br>0.0264*<br>0.2920<br>0.0748<br>0.1056<br>0.1053<br>0.5351	Est. -0.56 (SE=1.14)<br>Est. -0.52 (SE=1.17)<br>Est. -0.32 (SE=1.19)<br>Est. -0.50 (SE=1.17)<br>Est. -0.40 (SE=1.25)<br>Est. -0.27 (SE=1.18)<br>Est. -0.36 (SE=1.11)	0.0120*<br>0.0228*<br>0.1603<br>0.0293*<br>0.1012<br>0.2391<br>0.0954
Which factors do you use to preoperatively assess eloquent brain regions in your practice? (please select all that apply) *n=137*	• Anatomical location based on structural MRI	131 (95.6)	$_{a/b}$<0.0001*<br>$_{a/c}$<0.0001*<br>$_{a/d}$<0.0001*<br>$_{a/e}$<0.0001*<br>$_{a/f}$<0.0001*<br>$_{b/c}$<0.0001*<br>$_{b/d}$0.6943<br>$_{b/e}$<0.0001*<br>$_{b/f}$<0.0001*<br>$_{c/d}$<0.0001*<br>$_{c/e}$0.3011<br>$_{c/f}$0.0062*<br>$_{d/e}$<0.0001*<br>$_{d/f}$<0.0001*<br>$_{e/f}$0.0002*	1.10 (0.19 – 6.24)	0.9163	2.65 (0.47 – 15.11)	0.2719
	• Proximity to white matter tracts using DTI	93 (67.9)		2.54 (1.19 – 5.41)	0.0161*	0.36 (0.15 – 0.86)	0.0221*
	• Navigated Transcranial Magnetic Stimulation (nTMS)	15 (10.9)		1.95 (0.52 – 7.31)	0.3211	1.07 (0.34 – 3.30)	0.9109
	• Functional MRI (fMRI)	96 (70.1)		0.87 (0.39 – 1.93)	0.7270	3.68 (1.48 – 9.12)	0.0049*
	• Magnetoencephalography (MEG)	10 (7.3)		1.07 (0.26 – 4.37)	0.9218	3.49 (0.71 – 17.24)	0.1250
	• Eloquence classification systems or grading scales	32 (23.4)		1.22 (0.51 – 2.93)	0.6500	1.14 (0.46 – 2.84)	0.7743
	• None of the above	0 (0)		NA		NA	NA
	• Other						
	○ Neuropsychiatric testing	2 (1.5)		NA		NA	NA
	○ Physical exam as altered by tumor	1 (0.7)		NA		NA	NA
Do you use a classification system for grading eloquence? (please select all that apply) *n=137*	• Sawaya eloquence grading	17 (12.4)	$_{a/b}$<0.0001*<br>$_{a/c}$0.4879<br>$_{a/d}$0.0001*<br>$_{a/e}$0.0031*<br>$_{b/c}$<0.0001*<br>$_{b/d}$<0.0001*<br>$_{b/e}$<0.0001*<br>$_{c/d}$<0.0001*<br>$_{c/e}$0.0004*	1.02 (0.33 – 3.17)	0.9747	0.53 (0.16 – 1.79)	0.3088
	• Spetzler-Martin grading system for arteriovenous malformations	52 (38.0)		1.16 (0.41 – 3.32)	0.7788	2.85 (0.93 – 8.67)	0.0644
	• UCSF LGG-scale	21 (15.3)		0.86 (0.30 – 2.46)	0.7788	0.60 (0.19 – 1.89)	0.3841
	• Friedlein grading (FGA/B)	1 (0.7)		NA	NA	NA	NA
	• Shinoda topographical tumor staging system	4 (2.9)		NA	NA	NA	NA
	• None of the above	64 (46.7)		1.53 (0.73 – 3.18)	0.2559	0.60 (0.28 – 1.27)	0.1793
	• Other						

○ NTMS risk stratification	1 (0.7)	[d/e]0.1716	NA	NA	NA	NA
○ Our own one	1 (0.7)		NA	NA	NA	NA

Supplementary Table 2: Patient Cases

Case	Questions and response options	Overall response (%)	P-value	Adjusted OR for academic vs. non-academic and private practice (95% CI)	P-value	Adjusted OR for European vs. US neurosurgeons (95% CI)	P-value
1 *n=109*	Do you consider the location of the tumor:						
	• Eloquent	42 (38.5)[a]	$_{a/b}$0.0019*	1.46 (0.61 – 3.51)	0.3974	0.43 (0.18 – 1.03)	0.0577
	• Near eloquent	65 (59.6)[b]	$_{a/c}$<0.0001*	0.62 (0.26 – 1.48)	0.2787	2.10 (0.89 – 4.95)	0.0899
	• Not eloquent	2 (1.8)[c]	$_{b/c}$<0.0001*	NA	NA	NA	NA
	What would be your preferential surgical approach in this case?						
	• Biopsy	3 (2.8)[a]	$_{a/b}$1.0	NA	NA	NA	NA
	• Decompression/partial resection	3 (2.8)[b]	$_{a/c}$<0.0001*	NA	NA	NA	NA
	• Maximal safe resection with asleep mapping or monitoring	68 (62.4)[c]	$_{a/d}$<0.0001*	0.88 (0.37 – 2.09)	0.7723	1.56 (0.62 – 3.90)	0.3457
	• Maximal safe resection with awake mapping or monitoring	28 (24.8)[d]	$_{a/e}$0.1301	0.73 (0.29 – 1.87)	0.7333	0.31 (0.10 – 0.91)	0.0323*
	• Maximal safe resection without mapping or monitoring	8 (7.3)[e]	$_{a/f}$0.0792	2.96 (0.35 – 25.10)	0.3202	NA	NA
	• No surgery	0 (0)[f]	$_{b/c}$<0.0001*	NA	NA	NA	NA
			$_{b/d}$<0.0001*				
			$_{b/e}$0.1301				
			$_{b/f}$0.0792				
			$_{c/d}$<0.0001*				
			$_{c/e}$<0.0001*				
			$_{c/f}$<0.0001*				
			$_{d/e}$0.0004*				
			$_{e/f}$0.0041*				
2 *n=109*	Do you consider the location of the tumor:	51 (46.8)[a]	$_{a/b}$1.0	1.10 (0.48 – 2.53)	0.8300	0.39 (1.16 – 0.92)	0.0307*
	• Eloquent	51 (46.8)[b]	$_{a/c}$<0.0001*	1.10 (0.48 – 2.53)	0.8300	3.29 (1.37 – 7.89)	0.0077*
	• Near eloquent	7 (6.4)[c]	$_{b/c}$<0.0001*	0.50 (0.11 – 2.40)	0.3897	0.40 (0.07 – 2.32)	0.3081
	• Not eloquent						
	What would be your preferential surgical approach in this case?						
	• Biopsy	31 (28.4)[a]	$_{a/b}$<0.0001*	0.51 (0.21 – 1.24)	0.1374	0.71 (0.28 – 1.81)	0.4836
	• Decompression/partial resection	2 (1.8)[b]	$_{a/c}$0.1149	NA	NA	NA	NA
	• Maximal safe resection with asleep mapping or monitoring	42 (38.5)[c]	$_{a/d}$0.0013*	1.0 (0.42 – 2.33)	0.9808	2.86 (1.18 – 6.89)	0.0195*
	• Maximal safe resection with awake mapping or monitoring	12 (11)[d]	$_{a/e}$0.1158	NA	NA	0.18 (0.04 – 0.90)	0.0368*
	• Maximal safe resection without mapping or monitoring	21 (19.3)[e]	$_{a/f}$<0.0001*	0.75 (0.27 – 2.08)	0.5809	0.48 (0.14 – 1.60)	0.2324
	• No surgery	1 (0.9)[f]	$_{b/c}$<0.0001*	NA	NA	NA	NA
			$_{b/d}$0.0056*				

			$_{b/e}$<0.0001*				
			$_{b/f}$0.5657				
			$_{c/d}$<0.0001*				
			$_{c/e}$0.0018*				
			$_{c/f}$<0.0001*				
			$_{d/e}$0.0882				
			$_{d/f}$0.0017*				
			$_{e/f}$<0.0001*				
3 *n=109*	Do you consider the location of the tumor:						
	• Eloquent	55 (50.5)[a]	$_{a/b}$0.1739	1.35 (0.58 – 3.10)	0.4862	2.13 (0.91 – 4.98)	0.0812
	• Near eloquent	45 (41.3)[b]	$_{a/c}$<0.0001*	0.74 (0.35 – 1.71)	0.4845	0.48 (0.20 – 1.13)	0.0936
	• Not eloquent	9 (8.3)[c]	$_{b/c}$<0.0001*	0.78 (0.18 – 3.33)	0.7346	0.84 (0.16 – 4.43)	0.8415
	What would be your preferential surgical approach in this case?						
	• Biopsy	8 (7.3)[a]	$_{a/b}$0.7930	0.21 (0.05 – 0.93)	0.0401*	0.62 (0.13 – 2.93)	0.5433
	• Decompression/partial resection	7 (6.4)[b]	$_{a/c}$0.0004*	2.50 (0.29 – 21.67)	0.4056	0.85 (0.11 – 6.30)	0.8719
	• Maximal safe resection with asleep mapping or monitoring	27 (24.8)[c]	$_{a/d}$<0.0001*	0.73 (0.29 – 1.87)	0.5167	0.90 (0.36 – 2.27)	0.8192
	• Maximal safe resection with awake mapping or monitoring	44 (40.4)[d]	$_{a/e}$0.0036*	1.33 (0.56 – 3.16)	0.5130	1.59 (0.68 – 3.74)	0.2826
	• Maximal safe resection without mapping or monitoring	23 (21.1)[e]	$_{a/f}$0.0041*	1.56 (0.52 – 4.65)	0.4250	0.70 (0.23 – 2.14)	0.5370
	• No surgery	0 (0)[f]	$_{b/c}$0.0002*	NA	NA	NA	NA
			$_{b/d}$<0.0001*				
			$_{b/e}$0.0017*				
			$_{b/f}$0.0074*				
			$_{c/d}$0.0142				
			$_{c/e}$0.5169				
			$_{c/f}$<0.0001*				
			$_{d/e}$0.0021*				
			$_{d/f}$<0.0001*				
			$_{e/f}$<0.0001*				
4 *n=109*	Do you consider the location of the tumor:						
	• Eloquent	91 (83.5)[a]	$_{a/b}$<0.0001*	0.96 (0.31 – 2.97)	0.9456	0.81 (0.23 – 2.79)	0.7426
	• Near eloquent	13 (11.9)[b]	$_{a/c}$<0.0001*	0.88 (0.25 – 3.10)	0.8429	1.08 (0.27 – 4.30)	0.9180
	• Not eloquent	5 (4.6)[c]	$_{b/c}$0.0507	1.62 (0.17 – 15.11)	0.6712	1.74 (0.15 – 19.90)	0.6564
	What would be your preferential surgical approach in this case?						
	• Biopsy	2 (1.8)[a]	$_{a/b}$0.6232	NA	NA	NA	NA
	• Decompression/partial resection	3 (2.8)[b]	$_{a/c}$0.0056*	NA	NA	NA	NA

	• Maximal safe resection with asleep mapping or monitoring	12 (11)[c]	$_{a/d}$<0.0001*	1.21 (0.31 – 4.83)	0.7797	2.16 (0.52 – 8.97)	0.2880
	• Maximal safe resection with awake mapping or monitoring	84 (77.1)[d]	$_{a/e}$0.1462	1.25 (0.47 – 3.28)	0.6535	0.46 (0.16 – 1.35)	0.1586
	• Maximal safe resection without mapping or monitoring	6 (5.5)[e]	$_{a/f}$1.0	0.78 (0.14 – 4.51)	0.7851	0.85 (0.11 – 6.30)	0.8719
	• No surgery	2 (1.8)[f]	$_{b/c}$0.0172	NA	NA	NA	NA
			$_{b/d}$<0.0001*				
			$_{b/e}$0. 3187				
			$_{b/f}$1.0				
			$_{c/d}$<0.0001				
			$_{c/e}$0.1409				
			$_{c/f}$0.0056*				
			$_{d/e}$<0.0001*				
			$_{d/f}$<0.0001*				
			$_{e/f}$0.1462				
5 *n=108*	Do you consider the location of the tumor:						
	• Eloquent	17 (15.7)[a]	$_{a/b}$<0.0001*	0.48 (0.16 – 1.42)	0.1850	1.70 (0.52 – 5.57)	0.3771
	• Near eloquent	53 (49.1)[b]	$_{a/c}$0.0010*	2.46 (1.02 – 5.93)	0.0454*	0.63 (0.27 – 1.47)	0.2864
	• Not eloquent	38 (35.2)[c]	$_{b/c}$0.0390	0.62 (0.26 – 1.47)	0.2734	1.22 (0.50 – 2.96)	0.6597
	What would be your preferential surgical approach in this case?						
	• Biopsy	11 (10.2)[a]	$_{a/b}$0.0029	0.74 (0.20 – 2.68)	0.6495	1.35 (0.35 – 5.17)	0.6581
	• Decompression/partial resection	1 (0.9)[b]	$_{a/c}$<0.0001*	NA	NA	NA	NA
	• Maximal safe resection with asleep mapping or monitoring	44 (40.7)[c]	$_{a/d}$0.0111	1.27 (0.53 – 3.02)	0.5934	0.65 (2.28 – 1.50)	0.3114
	• Maximal safe resection with awake mapping or monitoring	25 (23.1)[d]	$_{a/e}$0.0111	0.99 (0.36 – 2.67)	0.9774	0.96 (0.35 – 2.66)	0.9388
	• Maximal safe resection without mapping or monitoring	25 (23.1)[e]	$_{a/f}$0.0107	0.99 (0.36 – 2.67)	0.9774	1.48 (0.52 – 4.27)	0.4642
	• No surgery	2 (1.9)[f]	$_{b/c}$<0.0001*	NA	NA	NA	NA
			$_{b/d}$<0.0001*				
			$_{b/e}$<0.0001*				
			$_{b/f}$0.5326				
			$_{c/d}$0.0056*				
			$_{c/e}$0.0056*				
			$_{c/f}$<0.0001*				
			$_{d/e}$1.0				
			$_{d/f}$<0.0001*				
			$_{e/f}$<0.0001*				
6 *n=108*	Do you consider the location of the tumor:						
	• Eloquent	23 (21.3)[a]	$_{a/b}$0.0659	0.65 (0.24 – 1.75)	0.3998	1.11 (0.39 – 3.16)	0.8379
	• Near eloquent	37 (34.3)[b]	$_{a/c}$0.0010*	0.58 (0.24 – 1.38)	0.2202	0.81 (0.34 – 1.97)	0.6449
	• Not eloquent	48 (44.4)[c]	$_{b/c}$0.1296	2.33 (0.95 – 5.73)	0.0645	1.12 (0.48 – 2.61)	0.7848

	What would be your preferential surgical approach in this case?						
	• Biopsy	5 (4.6)[a]	a/b 0.7408	0.24 (0.04 – 1.50)	0.1254	3.72 (0.40 – 34.72)	0.2488
	• Decompression/partial resection	4 (3.7)[b]	a/c <0.0001*	NA	NA	NA	NA
	• Maximal safe resection with asleep mapping or monitoring	37 (34.3)[c]	a/d 0.2695	1.06 (0.43 – 2.58)	0.8999	1.46 (0.61 – 3.52)	0.3973
	• Maximal safe resection with awake mapping or monitoring	9 (8.3)[d]	a/e <0.0001*	0.45 (0.11 – 1.79)	0.2535	0.41 (0.07 – 2.37)	0.3201
	• Maximal safe resection without mapping or monitoring	52 (48.1)[e]	a/f 0.0972	2.33 (0.97 – 5.63)	0.0591	0.54 (0.23 – 1.25)	0.1506
	• No surgery	1 (0.9)[f]	b/c <0.0001*	NA	NA	NA	NA
			b/d 0.1556				
			b/e <0.0001*				
			b/f 0.1709				
			c/d <0.0001*				
			c/e 0.0398				
			c/f <0.0001*				
			d/e <0.0001*				
			d/f 0.0096*				
			e/f <0.0001*				
7 *n=108*	Do you consider the location of the tumor:						
	• Eloquent	16 (14.8)[a]	a/b 0.0003*	0.43 (0.14 – 1.28)	0.1293	0.71 (0.22 – 2.32)	0.5712
	• Near eloquent	39 (36.1)[b]	a/c <0.0001*	2.29 (0.88 – 5.96)	0.0911	0.98 (0.41 – 2.35)	0.9678
	• Not eloquent	53 (49.1)[c]	b/c 0.0539	0.79 (0.34 – 1.84)	0.5833	1.21 (0.52 – 2.80)	0.6586
	What would be your preferential surgical approach in this case?						
	• Biopsy	3 (2.8)[a]	a/b 0.3013	NA	NA	NA	NA
	• Decompression/partial resection	1 (0.9)[b]	a/c 0.0003*	NA	NA	NA	NA
	• Maximal safe resection with asleep mapping or monitoring	19 (17.6)[c]	a/d <0.0001*	1.55 (0.47 – 5.11)	0.4734	0.98 (0.34 – 2.82)	0.9657
	• Maximal safe resection with awake mapping or monitoring	29 (26.9)[d]	a/e <0.0001*	1.01 (0.39 – 2.26)	0.9785	2.51 (0.91 – 6.90)	0.0753
	• Maximal safe resection without mapping or monitoring	56 (51.9)[e]	a/f 0.0806	0.92 (0.40 – 2.14)	0.8485	0.43 (0.18 – 1.02)	0.0562
	• No surgery	0 (0)[f]	b/c <0.0001*	NA	NA	NA	NA
			b/d <0.0001*				
			b/e <0.0001*				
			b/f 0.3242				
			c/d 0.1012				
			c/e <0.0001*				
			c/f <0.0001*				
			d/e 0.0002*				
			d/f <0.0001*				
			e/f <0.0001*				

8 *n=108*	Do you consider the location of the tumor:						
	• Eloquent	29 (26.9)[a]	a/b <0.0001*	0.52 (0.21 – 1.29)	0.1570	0.65 (0.25 – 1.72)	0.3893
	• Near eloquent	59 (54.6)[b]	a/c 0.1415	1.29 (0.56 – 3.01)	0.5494	1.85 (0.79 – 4.35)	0.1568
	• Not eloquent	20 (18.5)[c]	b/c <0.0001*	1.68 (0.51 – 5.50)	0.3933	0.62 (0.21 – 1.85)	0.3943
	What would be your preferential surgical approach in this case?						
	• Biopsy	10 (9.3)[a]	a/b 0.0456	0.89 (0.21 – 3.68)	0.8692	0.67 (0.17 – 2.68)	0.5712
	• Decompression/partial resection	3 (2.8)[b]	a/c <0.0001*	NA	NA	NA	NA
	• Maximal safe resection with asleep mapping or monitoring	58 (53.7)[c]	a/d 0. 6146	1.23 (0.53 – 2.86)	0.6324	1.40 (0.60 – 3.27)	0.4318
	• Maximal safe resection with awake mapping or monitoring	8 (7.4)[d]	a/e 0.0014*	0.62 (0.14 – 2.76)	0.5267	2.73 (0.27 – 27.29)	0.3932
	• Maximal safe resection without mapping or monitoring	28 (25.9)[e]	a/f 0.0051*	1.21 (0.45 – 3.23)	0.7032	0.65 (0.25 – 1.72)	0.3893
	• No surgery	1 (0.9)[f]	b/c <0.0001*	NA	NA	NA	NA
			b/d 0.1253				
			b/e <0.0001*				
			b/f 0.3013				
			c/d <0.0001*				
			c/e <0.0001*				
			c/f <0.0001*				
			d/e 0.0003*				
			d/f 0.0169				
			e/f <0.0001*				
9 *n=108*	Do you consider the location of the tumor:						
	• Eloquent	42 (38.9)	a/b 0.2726	0.44 (0.19 – 1.03)	0.0590	1.31 (0.55 – 3.12)	0.5443
	• Near eloquent	50 (46.3)	a/c 0.0001*	2.11 (0.87 – 5.07)	0.0971	0.85 (0.37 – 1.96)	0.7006
	• Not eloquent	16 (14.8)	b/c <0.0001*	1.18 (0.35 – 4.0)	0.7883	0.85 (0.27 – 2.67)	0.7805
	What would be your preferential surgical approach in this case?						
	• Biopsy	7 (6.5)	a/b 0.1977	0.49 (0.10 – 2.32)	0.3655	1.81 (0.31 – 10.46)	0.5052
	• Decompression/partial resection	3 (2.8)	a/c 0.0003*	NA	NA	NA	NA
	• Maximal safe resection with asleep mapping or monitoring	26 (24.1)	a/d <0.0001*	0.83 (0.31 – 2.17)	0.6962	0.95 (0.35 – 2.53)	0.9138
	• Maximal safe resection with awake mapping or monitoring	35 (32.4)	a/e <0.0001*	1.46 (0.57 – 3.71)	0.4306	0.82 (0.33 – 2.01)	0.6616
	• Maximal safe resection without mapping or monitoring	37 (34.3)	a/f 0.0072*	0.48 (0.20 – 1.14)	0.0950	1.09 (0.45 – 2.63)	0.8429
	• No surgery	0 (0)	b/c <0.0001*	NA	NA	NA	NA
			b/d <0.0001*				
			b/e <0.0001*				
			b/f 0.0806				
			c/d 0.1765				
			c/e 0.0969				

			$_{c/f}$<0.0001*				
			$_{d/e}$0.7676				
			$_{d/f}$<0.0001*				
			$_{e/f}$<0.0001*				
10 *n=107*	Do you consider the location of the tumor:						
	• Eloquent	70 (65.4)	$_{a/b}$<0.0001*	0.93 (0.38 – 2.26)	0.8657	1.09 (0.44 – 2.71)	0.8455
	• Near eloquent	34 (31.8)	$_{a/c}$<0.0001*	1.12 (0.45 – 2.80)	0.8055	0.83 (0.32 – 2.12)	0.6948
	• Not eloquent	3 (2.8)	$_{b/c}$<0.0001*	0.77 (0.07 – 8.86)	0.8363	NA	NA
	What would be your preferential surgical approach in this case?						
	• Biopsy	15 (14)	$_{a/b}$0.3537	1.08 (0.32 – 3.71)	0.8986	0.71 (0.22 – 2.32)	0.5712
	• Decompression/partial resection	20 (18.7)	$_{a/c}$0.8358	0.67 (0.24 – 1.88)	0.4439	1.95 (0.61 – 6.25)	0.2637
	• Maximal safe resection with asleep mapping or monitoring	16 (15)	$_{a/d}$0.0001*	1.83 (0.48 – 6.94)	0.3752	0.49 (0.15 – 1.64)	0.2481
	• Maximal safe resection with awake mapping or monitoring	40 (37.4)	$_{a/e}$1.0	0.86 (0.36 – 2.04)	0.7270	0.88 (0.37 – 2.06)	0.7622
	• Maximal safe resection without mapping or monitoring	15 (14)	$_{a/f}$0.0003*	1.66 (0.43 – 6.36)	0.4585	1.62 (0.44 – 5.98)	0.4702
	• No surgery	1 (0.9)	$_{b/c}$0.4707	NA	NA	NA	NA
			$_{b/d}$0.0024*				
			$_{b/e}$0.3537				
			$_{b/f}$<0.0001*				
			$_{c/d}$0.0002*				
			$_{c/e}$0.8358				
			$_{c/f}$0.0001*				
			$_{d/e}$0.0001*				
			$_{d/f}$<0.0001*				
			$_{e/f}$0.0003*				
11	Do you consider the location of the tumor: *n=107*						
	• Eloquent	9 (8.4)	$_{a/b}$<0.0001*	0.76 (0.18 – 3.26)	0.7124	1.51 (0.34 – 6.74)	0.5908
	• Near eloquent	45 (42.1)	$_{a/c}$<0.0001*	0.77 (0.33 – 1.80)	0.5469	0.66 (0.28 – 1.54)	0.3340
	• Not eloquent	53 (49.5)	$_{b/c}$0.2784	1.41 (0.60 – 3.31)	0.4243	1.32 (0.57 – 3.05)	0.5218
	What would be your preferential surgical approach in this case? *n=106*						
	• Biopsy	4 (3.7)	$_{a/b}$0.7111	NA	NA	NA	NA
	• Decompression/partial resection	3 (2.8)	$_{a/c}$<0.0001*	NA	NA	NA	NA
	• Maximal safe resection with asleep mapping or monitoring	38 (35.5)	$_{a/d}$0.0974	1.44 (0.58 – 3.57)	0.4313	2.24 (0.92 – 5.42)	0.0749
	• Maximal safe resection with awake mapping or monitoring	10 (9.3)	$_{a/e}$<0.0001*	0.56 (0.15 – 2.13)	0.3932	0.27 (0.03 – 2.69)	0.2628
	• Maximal safe resection without mapping or monitoring	49 (45.8)	$_{a/f}$0.4259	1.73 (0.73 – 4.12)	0.2172	0.50 (0.21 – 1.18)	0.1140
	• No surgery	2 (1.9)	$_{b/c}$ <0.0001*	NA	NA	NA	NA
			$_{b/d}$0.0466				
			$_{b/e}$<0.0001*				

			$_{b/f}$0.6646<br>$_{c/d}$<0.0001*<br>$_{c/e}$0.1260<br>$_{c/f}$<0.0001*<br>$_{d/e}$<0.0001*<br>$_{d/f}$0.0188<br>$_{e/f}$<0.0001*				
12 *n=106*	Do you consider the location of the tumor:						
	• Eloquent	9 (8.4)	$_{a/b}$<0.0115	1.42 (0.28 – 7.26)	0.6734	0.48 (0.11 – 2.14)	0.3334
	• Near eloquent	22 (20.6)	$_{a/c}$<0.0001*	0.48 (0.18 – 1.29)	0.1450	0.60 (0.20 – 1.80)	0.3642
	• Not eloquent	75 (70.1)	$_{b/c}$<0.0001*	1.62 (0.66 – 3.99)	0.2934	1.99 (0.77 – 5.17)	0.1567
	What would be your preferential surgical approach in this case?						
	• Biopsy	1 (0.9)	$_{a/b}$0.5345	NA	NA	NA	NA
	• Decompression/partial resection	2 (1.9)	$_{a/c}$<0.0001*	NA	NA	NA	NA
	• Maximal safe resection with asleep mapping or monitoring	29 (27.1)	$_{a/d}$0.0928	1.05 (0.41 – 2.72)	0.9200	1.12 (0.44 – 2.85)	0.8143
	• Maximal safe resection with awake mapping or monitoring	5 (4.7)	$_{a/e}$<0.0001*	1.61 (0.17 – 15.03)	0.6755	2.66 (0.27 – 26.62)	0.4054
	• Maximal safe resection without mapping or monitoring	68 (63.6)	$_{a/f}$1.0	0.86 (0.35 – 2.09)	0.7345	0.71 (0.29 – 1.71)	0.4455
	• No surgery	1 (0.9)	$_{b/c}$ <0.0001*	NA	NA	NA	NA
			$_{b/d}$0.2527<br>$_{b/e}$<0.0001*<br>$_{b/f}$0.5345<br>$_{c/d}$<0.0001*<br>$_{c/e}$<0. 0001*<br>$_{c/f}$<0.0001*<br>$_{d/e}$<0.0001*<br>$_{d/f}$0.0928<br>$_{e/f}$<0.0001*				

Supplementary Table 3: Survey responses specified by surgeon's experience with glioma resections

Question	Response options	<100 glioma resections performed 33(%)	100-500 glioma resections performed 51(%)	>500 glioma resections performed 51(%)	*P* value
In your opinion, is there a need for a consensus definition of eloquent brain regions in neurosurgery? *n*=135	• Yes<br>• No	27 (81.8)<br>6 (18.2)	29 (56.9)<br>22 (43.1)	36 (70.6)<br>15 (29.4)	0.0505
Are awake craniotomies performed at your institution? *n*=135	• Yes<br>• No	24 (72.7)<br>9 (27.3)	43 (84.3)<br>8 (15.7)	48 (94.1)<br>3 (5.9)	0.0258*
Are intra-operative asleep mapping techniques performed at your institution? (Evoked potentials (MEP/SSEP) with subdural grid/strip electrodes or continuous dynamic mapping (CDM) on suction tube or CUSA) *n*=135	• Yes<br>• No	25 (75.8)<br>8 (24.2)	42 (82.4)<br>9 (17.6)	46 (90.2)<br>5 (9.8)	0.2048
Please indicate on a Likert scale of 1-5 for each given brain structure whether or not you would classify it as eloquent.	• Motor cortex *(n=135)*	Mean 4.8 (SD = 0.64)	Mean 4.7 (SD = 1.09)	Mean 4.9 (SD = 0.56)	NA
	• Language cortex *(n=135)*	Mean 4.7 (SD = 0.72)	Mean 4.6 (SD = 1.11)	Mean 4.9 (SD = 0.57)	
	• Sensory cortex *(n=135)*	Mean 4.2 (SD = 1.06)	Mean 4.0 (SD = 1.30)	Mean 4.3 (SD = 0.98)	
	• Visual cortex *(n=135)*	Mean 4.5 (SD = 0.75)	Mean 4.2 (SD = 1.27)	Mean 4.6 (SD = 0.88)	
	• Basal ganglia *(n=135)*	Mean 4.5 (SD = 0.87)	Mean 4.0 (SD = 1.26)	Mean 4.1 (SD = 1.14)	
	• Insula *(n=135)*	Mean 3.8 (SD = 1.30)	Mean 3.7 (SD = 1.16)	Mean 3.9 (SD = 0.99)	
	• Premotor cortex *(n=135)*	Mean 3.5 (SD = 1.23)	Mean 3.4 (SD = 1.12)	Mean 3.4 (SD = 0.94)	
	• Supplementary motor cortex (SMA) *(n=135)*	Mean 3.6 (SD = 1.12)	Mean 3.5 (SD = 1.19)	Mean 3.6 (SD = 1.08)	
	• Hippocampus & parahippocampal area *(n=134)*	Mean 3.5 (SD = 1.25)	Mean 3.1 (SD = 1.25)	Mean 3.5 (SD = 1.02)	
	• Internal capsule *(n=134)*	Mean 4.8 (SD = 0.65)	Mean 4.4 (SD = 1.18)	Mean 4.9 (SD = 0.48)	
	• Dominant arcuate fasciculus *(n=134)*	Mean 4.4 (SD = 0.86)	Mean 4.2 (SD = 1.35)	Mean 4.6 (SD = 0.83)	
	• Dominant inferior fronto-occipital fasciculus *(n=134)*	Mean 3.6 (SD = 1.17)	Mean 3.7 (SD = 1.29)	Mean 4.0 (SD = 0.92)	
	• Corpus callosum *(n=134)*	Mean 3.1 (SD = 1.20)	Mean 3.0 (SD = 1.13)	Mean 2.9 (SD = 1.02)	
	• Corona radiata *(n=134)*	Mean 3.5 (SD = 1.18)	Mean 3.6 (SD = 1.34)	Mean 3.6 (SD = 1.25)	
	• Optic radiation *(n=134)*	Mean 4.1 (SD = 0.78)	Mean 3.8 (SD = 1.25)	Mean 4.1 (SD = 0.98)	
	• Meyer's loop *(n=134)*	Mean 4.0 (SD = 0.88)	Mean 3.4 (SD = 1.13)	Mean 3.9 (SD = 0.92)	
	• Superior longitudinal fasciculus *(n=134)*	Mean 3.4 (SD = 1.27)	Mean 3.3 (SD = 1.35)	Mean 3.7 (SD = 0.99)	
	• Inferior longitudinal fasciculus *(n=134)*	Mean 3.4 (SD = 1.22)	Mean 3.2 (SD = 1.34)	Mean 3.5 (SD = 0.88)	
	• Middle longitudinal fasciculus *(n=134)*	Mean 3.2 (SD = 1.27)	Mean 3.0 (SD = 1.28)	Mean 3.2 (SD = 0.94)	

	• Frontal aslant tract *(n=134)*	Mean 3.3 (SD = 1.16)	Mean 3.1 (SD = 1.34)	Mean 3.4 (SD = 1.00)	
	• Uncinate fasciculus *(n=134)*	Mean 3.2 (SD = 1.34)	Mean 3.1 (SD = 1.32)	Mean 3.3 (SD = 1.03)	
Which factors do you use to preoperatively assess eloquent brain regions in your practice? (please select all that apply) *n=137*	• Anatomical location based on structural MRI	31 (93.9)	49 (94.2)	51 (100)	0.2102
	• Proximity to white matter tracts using DTI	24 (72.7)	29 (55.8)	40 (78.4)	0.0388*
	• Navigated Transcranial Magnetic Stimulation (nTMS)	3 (9.1)	4 (7.7)	8 (15.7)	0.3978
	• Functional MRI (fMRI)	26 (78.8)	34 (65.4)	36 (70.6)	0.4175
	• Magnetoencephalography (MEG)	1 (3.0)	3 (5.8)	6 (11.8)	0.2789
	• Eloquence classification systems or grading scales	7 (21.2)	11 (21.2)	12 (23.5)	0.9500
	• None of the above	0 (0)	0 (0)	0 (0)	NA
	• Other	0 (0)	2 (3.8)	1 (2.0)	0.4948
Do you use a classification system for grading eloquence? (please select all that apply) *n=137*	• Sawaya eloquence grading	4 (12.1)	7 (13.5)	6 (11.8)	0.9639
	• Spetzler-Martin grading system for arteriovenous malformations	20 (60.6)	16 (30.8)	16 (31.4)	0.0099*
	• UCSF LGG-scale	0 (0)	10 (19.2)	11 (21.6)	0.0177*
	• Friedlein grading (FGA/B)	1 (3.0)	0 (0)	0 (0)	0.2076
	• Shinoda topographical tumor staging system	2 (6.1)	0 (0)	2 (3.9)	0.2378
	• None of the above	10 (30.3)	25 (48.1)	29 (56.9)	0.0576
	• Other	1 (3.0)	0 (0)	1 (2.0)	0.4929

Supplementary Table 4: Survey responses specified by surgeon's experience with awake craniotomies

Question	Response options	<50 awake craniotomies performed 67(%)	50-100 awake craniotomies performed 40(%)	100-500 awake craniotomies performed 22(%)	>500 awake craniotomies performed 6(%)	*P* value
In your opinion, is there a need for a consensus definition of eloquent brain regions in neurosurgery? *n*=135	• Yes<br>• No	49 (73.1)<br>18 (26.9)	25 (62.5)<br>15 (37.5)	15(68.2)<br>7 (31.8)	3 (50)<br>3 (50)	0.5191
Are awake craniotomies performed at your institution? *n*=135	• Yes<br>• No	50 (74.6)<br>17 (25.4)	37 (92.5)<br>3 (7.5)	22 (100)<br>0 (0)	6 (100)<br>0 (0)	0.0315*
Are intra-operative asleep mapping techniques performed at your institution? (Evoked potentials (MEP/SSEP) with subdural grid/strip electrodes or continuous dynamic mapping (CDM) on suction tube or CUSA) *n*=135	• Yes<br>• No	51 (76.1)<br>16 (23.9)	37 (92.5)<br>3 (7.5)	19 (86.4)<br>3 (13.6)	6 (100)<br>0 (0)	0.1689
Please indicate on a Likert scale of 1-5 for each given brain structure whether or not you would classify it as eloquent.	• Motor cortex *(n=135)*	Mean 4.8 (SD = 0.82)	Mean 5.0 (SD = 0.00)	Mean 5.0 (SD = 0.00)	Mean 5.0 (SD = 0.00)	NA
	• Language cortex *(n=135)*	Mean 4.7 (SD = 0.87)	Mean 4.9 (SD = 0.27)	Mean 5.0 (SD = 0.00)	Mean 5.0 (SD = 0.00)	
	• Sensory cortex *(n=135)*	Mean 4.1 (SD = 1.14)	Mean 4.3 (SD = 1.15)	Mean 4.6 (SD = 0.62)	Mean 4.5 (SD = 0.84)	
	• Visual cortex *(n=135)*	Mean 4.5 (SD = 0.92)	Mean 4.7 (SD = 0.79)	Mean 4.6 (SD = 0.70)	Mean 4.7 (SD = 0.82)	
	• Basal ganglia *(n=135)*	Mean 4.3 (SD = 1.10)	Mean 4.5 (SD = 0.81)	Mean 3.9 (SD = 1.06)	Mean 3.8 (SD = 1.17)	
	• Insula *(n=135)*	Mean 3.7 (SD = 1.15)	Mean 4.0 (SD = 0.79)	Mean 3.8 (SD = 0.94)	Mean 4.7 (SD = 0.52)	
	• Premotor cortex *(n=135)*	Mean 3.3 (SD = 1.00)	Mean 4.0 (SD = 0.87)	Mean 3.3 (SD = 0.89)	Mean 3.2 (SD = 0.75)	
	• Supplementary motor cortex (SMA) *(n=135)*	Mean 3.3 (SD = 1.00)	Mean 4.2 (SD = 0.94)	Mean 3.5 (SD = 1.04)	Mean 3.7 (SD = 0.82)	
	• Hippocampus & parahippocampal area *(n=134)*	Mean 3.4 (SD = 1.19)	Mean 3.7 (SD = 0.94)	Mean 3.3 (SD = 1.03)	Mean 2.7 (SD = 1.51)	
	• Internal capsule *(n=134)*	Mean 4.7 (SD = 0.84)	Mean 4.8 (SD = 0.47)	Mean 4.9 (SD = 0.47)	Mean 4.8 (SD = 0.41)	
	• Dominant arcuate fasciculus *(n=134)*	Mean 4.4 (SD = 0.96)	Mean 4.7 (SD = 0.61)	Mean 4.6 (SD = 1.04)	Mean 4.8 (SD = 0.41)	
	• Dominant inferior fronto-occipital fasciculus *(n=134)*	Mean 3.6 (SD = 1.17)	Mean 4.3 (SD = 0.68)	Mean 4.2 (SD = 1.11)	Mean 3.8 (SD = 1.17)	
	• Corpus callosum *(n=134)*	Mean 3.1 (SD = 1.23)	Mean 3.4 (SD = 0.96)	Mean 2.6 (SD = 0.78)	Mean 2.0 (SD = 1.10)	
	• Corona radiata *(n=134)*	Mean 3.5 (SD = 1.33)	Mean 4.1 (SD = 1.12)	Mean 3.7 (SD = 1.33)	Mean 4.5 (SD = 0.84)	
	• Optic radiation *(n=134)*	Mean 4.1 (SD = 0.98)	Mean 4.3 (SD = 0.80)	Mean 4.0 (SD = 1.08)	Mean 4.2 (SD = 1.17)	
	• Meyer's loop *(n=134)*	Mean 3.9 (SD = 0.95)	Mean 4.1 (SD = 0.86)	Mean 3.8 (SD = 1.06)	Mean 3.7 (SD = 1.03)	
	• Superior longitudinal fasciculus *(n=134)*	Mean 3.3 (SD = 1.34)	Mean 3.8 (SD = 0.93)	Mean 3.7 (SD = 1.07)	Mean 4.5 (SD = 1.22)	
	• Inferior longitudinal fasciculus *(n=134)*	Mean 3.4 (SD = 1.27)	Mean 3.6 (SD = 0.91)	Mean 3.4 (SD = 1.04)	Mean 3.8 (SD = 1.17)	
	• Middle longitudinal fasciculus *(n=134)*	Mean 3.1 (SD = 1.32)	Mean 3.4 (SD = 0.96)	Mean 3.2 (SD = 1.22)	Mean 3.0 (SD = 1.10)	

	• Frontal aslant tract *(n=134)*<br>• Uncinate fasciculus *(n=134)*	Mean 3.1 (SD = 1.15)<br>Mean 3.1 (SD = 1.30)	Mean 3.7 (SD = 1.18)<br>Mean 3.6 (SD = 1.04)	Mean 3.3 (SD = 1.19)<br>Mean 3.3 (SD = 1.19)	Mean 3.8 (SD = 1.17)<br>Mean 3.8 (SD.= 1.17)	
Which factors do you use to preoperatively assess eloquent brain regions in your practice? (please select all that apply) *n=137*	• Anatomical location based on structural MRI	41 (95.3)	25 (96.2)	18 (100)	6 (85.7)	0.4653
	• Proximity to white matter tracts using DTI	26 (60.5)	22 (84.6)	16 (88.9)	6 (85.7)	0.0406*
	• Navigated Transcranial Magnetic Stimulation (nTMS)	5 (11.6)	3 (11.5)	4 (22.2)	0 (0)	0.4654
	• Functional MRI (fMRI)	29 (67.4)	21 (80.8)	10 (55.6)	5 (71.4)	0.3504
	• Magnetoencephalography (MEG)	3 (7)	0 (0)	4 (22.2)	0 (0)	0.0389*
	• Eloquence classification systems or grading scales	13 (30.2)	9 (34.6)	1 (5.6)	0 (0)	0.0466*
	• None of the above	0 (0)	0 (0)	0 (0)	0 (0)	NA
	• Other	1 (2.3)	1 (3.8)	1 (5.6)	0 (0)	0.8740
Do you use a classification system for grading eloquence? (please select all that apply) *n=137*	• Sawaya eloquence grading	6 (9.3)	4 (15.4)	1 (5.6)	0 (0)	0.5443
	• Spetzler-Martin grading system for arteriovenous malformations	24 (55.8)	8 (30.8)	2 (11.1)	0 (0)	0.0009*
	• UCSF LGG-scale	3 (7)	6 (23.1)	3 (16.7)	1 (14.3)	0.2978
	• Friedlein grading (FGA/B)	1 (2.3)	0 (0)	0 (0)	0 (0)	0.7533
	• Shinoda topographical tumor staging system	2 (4.7)	2 (7.7)	0 (0)	0 (0)	0.5974
	• None of the above	14 (32.6)	13 (50)	15 (83.3)	5 (71.4)	0.0023*
	• Other	2 (4.7)	0 (0)	0 (0)	0 (0)	0.4892
